# The pathology of failed McKee-Farrar implants: correlation with modern metal-on-metal-implant failure

**DOI:** 10.1007/s10856-017-5882-y

**Published:** 2017-03-22

**Authors:** Mitsuru Munemoto, George Grammatopoulos, Yasuhito Tanaka, Max Gibbons, Nicholas A. Athanasou

**Affiliations:** 1 Nuffield Department Orthopaedics Rheumatology & Musculoskeletal Sciences (NDORMS), University of Oxford, Windmill Road, Oxford, UK; 2 Dept of Orthopaedic Surgery, Nara medical University, Kashihara-City, 634-8522 Nara Japan; 3 Nuffield Orthopaedic Centre, Windmill Road, Headington, Oxford, OX3 7LD UK

## Abstract

**Abstract:**

The McKee-Farrar (MF) prosthesis was the first widely used total hip replacement (THR) to employ a metal-on-metal (MoM) articulation. These implants had a high rate of early aseptic loosening but a number achieved good long-term implant survival, stimulating the reintroduction of second and third generation implants of this type. In this study we analysed archival histopathology of periprosthetic tissues in twenty cases of MF aseptic implant failure to determine if there was evidence of an innate and adaptive immune response similar to that seen in modern MoM implants. The presence of macrophages, the extent of necrosis and the ALVAL response were graded semi-quantitatively. Variable but in most cases extensive tissue necrosis was associated with a heavy macrophage response to Cobalt-Chrome (Co-Cr) wear particles in periprosthetic tissues; most cases also contained evidence of a predominantly lymphocyte response which in eight cases was moderate or heavy (Oxford Grade 2/3). Our findings show that inflammatory and necrotic changes to deposition of Co-Cr wear particles are found in periprosthetic tissues of failed MF implants, indicating that there is an innate and adaptive response similar to that noted in second/third generation MoM implants; they also suggest that the pathobiological response to metal wear particles is likely to have contributed to MF implant failure in these cases.

**Graphical Abstract:**

## Introduction

G.K. McKee and J Watson-Farrar developed a metal on metal (MoM) hip joint prosthesis for total hip replacement (THR) in the 1960s [1–4]. Large numbers of these metal-on-metal (MoM) implants were implanted but they eventually fell out of favour when superior outcomes were reported with the low-friction Charnley metal on polyethylene (MoP) prosthesis. It was noted that McKee-Farrar (MF) hip replacements had a high rate of aseptic loosening in the first 10 years; McKee stopped using the all-metal system in 1972 [1]. Failure of MF implants was thought to be due mainly to poor implant design [1, 5]. The stem of the femoral component was curved with narrow medial and lateral borders and had multiple sharp corners [1, 6]. These features resulted in areas of high localised stress within the cement mantle; the broad neck of the MF femoral component was also prone to impingement against the metal rim of the acetabular component. The original surgical technique for implantation did not include reaming and routine deepening of the acetabulum, and, as a result, the components did not have adequate lateral bone cover [1, 6]. Despite these shortcomings, some MF hip replacements have shown impressive survival outcomes with several lasting more than 20 years [5–10].

The relative success of some MF and other first-generation MoM implant designs stimulated the drive to develop second- and third-generation MoM hip implants [11]. The modern MoM implants are composed of a cobalt-chrome molybdenum alloy similar to that used in the MF implant. Although some second and third-generation MoM designs have performed reasonably, a number have been associated with implant failure [12–14]. This is considered to be due in large measure to the pathobiological response to deposition of cobalt–chrome (Co–Cr) wear particles in periprosthetic tissues in which extensive necrosis an innate and foreign body macrophage response to Co–Cr wear is often seen as well as prominent lymphoid infiltrate which is characteristically perivascular in distribution; this has been termed ALVAL (aseptic lymphocyte-dominated vasculitis-associated lesion) and is believed to reflect the adaptive immune response to Co–Cr wear in periprosthetic tissues [13, 15–21].

There are relatively few reports on the histopathology of failed MF hip implants; some of these noted pathological changes similar to those reported in second- and third-generation MoM hip implants [22–27]. With one exception [22], these studies examined only a handful of cases. In this study, we have analysed histopathological findings in periprosthetic tissues around twenty failed MF hip implants with regard to the inflammatory and necrotic changes known to be associated with second- and third-generation MoM hip implants. Our aim has been to determine whether Co-Cr particles from MF implants induced a similar pathobiological response in periprosthetic tissues, particularly with regard to the necrotic and inflammatory changes that reflect the innate (non-specific) and adaptive (specific) host immune response to deposition of Co–Cr wear particles.

## Materials and methods

Periprosthetic tissue histology from twenty cases of failed MF hip implants undergoing revision arthroplasty for aseptic loosening were obtained from the files of the Histopathology Department, Nuffield Orthopaedic Centre, Oxford. The cases studied included 15 females and 5 males; the age range of the patients was 55 to 84 years. The original hip arthroplasty procedure was carried out between 1968 and 1974 and revision surgery was carried out between 1975 and 2008. There was limited clinical information and radiology for most cases was not available. Implant duration was 2 to 41 years. Clinical information provided on the pathology forms noted that the specimens were associated with “failure” of an MF hip implant: eleven of the cases referred to “loosening” of an MF implant. Two cases of sepsis were excluded. Details of patient age and implant duration are shown in Table 1.

**Table 1:** Case details and pathological findings in 20 failed McKee–Farrar hip implants

Case details				Pathological findings		
Case No.	Sex	Age at revision	Implant duration (year)	Necrosis	Macrophage	Oxford ALVAL score
1	F	73	3	3	3	3
2	F	71	3	3	3	3
3	M	39	3	3	3	0
4	F	44	4	3	3	3
5	F	63	4	3	3	0
6	F	84	4	2	2	1
7	F	68	5	3	2	1
8	F	72	5	3	3	3
9	F	83	5	3	2	1
10	F	85	7	2	3	2
11	M	62	7	3	3	1
12	F	70	9	3	3	2
13	M	73	9	3	3	3
14	M	55	14	3	1	0
15	F	84	25	3	3	1
16	F	61	27	3	3	1
17	F	78	28	3	3	1
18	M	68	30	3	3	1
19	F	57	33	3	3	1
20	F	74	41	3	3	2

The tissues analysed were the joint capsule in all cases and the femoral and/or acetabular membrane in 10 cases. The tissues were fixed in 10% formalin, embedded in paraffin wax, sectioned and stained with haematoxylin-eosin. Tissue necrosis and the extent of the inflammatory cell infiltrate in MoM periprosthetic tissues was assessed semi-quantitatively by histology as previously described [16, 17]. The number of macrophages was scored as 0 (absent), 1+ (few), 2+ (many), 3+ (abundant); necrosis was scored as 0 (absent), 1+ (scattered small necrotic areas), 2+ (frequent small or large necrotic areas: up to 25% tissue involvement), 3+ (extensive necrosis: more than 25% tissue involvement). The perivascular lymphoid infiltrate was graded using the Oxford ALVAL scoring system [16], this has been shown to correlate well with the Campbell scoring system [28]. In the Oxford System, grade 0 = no evidence of perivascular lymphocyte infiltrate; grade 1 = little evidence of a perivascular lymphocytic infiltrate, with lymphocyte cuffing of vessels being fewer than five cells in thickness; grade 2 = several perivascular lymphoid aggregates, with lymphocytic cuffing of vessels being five to ten cells in thickness; and grade 3 = numerous large perivascular lymphoid aggregates, with lymphocyte cuffing around vessels being more than ten cells in thickness.

Immunohistochemistry was carried out using an indirect immunoperoxidase technique to determine the antigenic phenotype of phagocytic macrophages and lymphoid cells in the inflammatory infiltrate of periprosthetic tissues. The list of monoclonal antibodies used in this study and their antigen specificity is shown in Table 2.

**Table 2:** Antigen specificity of monoclonal antibodies used in this study

Antibody (clone)	Antigen specificity	Source
F7.2.38	CD3: T cells	Dakopatts
NCL-L-CD4-368	CD4: T helper cells	Novocastra
CR/144B	CD8: T suppressor cells	Dakopatts
CD14-223	CD14: monocytes macrophages	Novocastra
L26	CD20: B cells	Dakopatts
KP1	CD68: monocytes macrophages	Dakopatts
CR3/43	HLA-DR	Dakopatts
124	BCL-2 oncoprotein: apoptosis	Dakopatts
NCL-CD163	CD163: macrophages	Novocastra

## Results

**Fig. 1:**
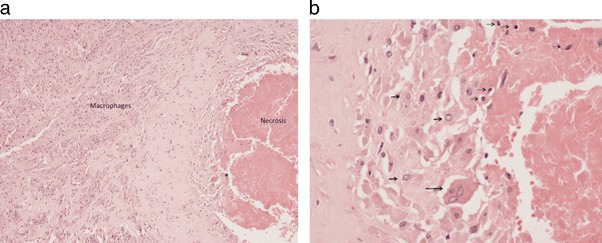
**a** Histology of periprosthetic fibrous tissue membrane from a failed MF implant showing necrosis on the surface with underlying foreign body macrophage infiltrate. **b** High power view of area of surface necrosis in 1a. showing a viable giant cell and macrophages with vesicular nuclei (some marked by thick *arrows*); phagocytosed aggregates of metal wear particles are evident as a *black* dot in the cytoplasm. There are also apoptotic macrophages with pyknotic nuclei (some marked by thin *arrows*)

**Fig. 2:**
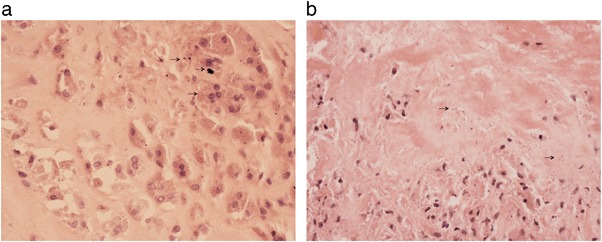
Histology of periprosthetic tissue from a failed MF implant showing: **a** viable macrophages with phagocytosed (*brown-black*) Co–Cr wear particles of variable size (some arrowed) **b** necrotic and apoptotic macrophages with released wear particles (some arrowed) (color figure online)

The most notable feature seen in the joint capsule and periprosthetic fibrous tissue membrane of all MF implant failures was a variable but generally extensive degree of surface tissue necrosis associated with an underlying heavy foreign body macrophage infiltrate (Fig. 1a). The tissues had a prominent zone of surface necrosis in which there were viable macrophages and occasional giant cells containing Co-Cr particles; some macrophages showed changes of apoptosis such as nuclear pyknosis (Fig. 1b). Beneath this zone there was a prominent macrophage infiltrate. Viable macrophages had vesicular nuclei and discrete plump cytoplasm which contained granular brown or black particles, indicating Co–Cr wear particle phagocytosis (Fig. 2a). On the surface, there was macrophage necrosis and apoptosis, evident as loss of cytoplasmic outline, nuclear pyknosis and release of wear particles into the degenerate tissue matrix (Fig. 2b). Histological findings regarding the extent of necrosis and macrophage infiltration are summarised in Table 1. A heavy eosinophil polymorph infiltrate was noted in two specimens. A few neutrophil polymorphs (less than 1 per high power field on average) were noted in two specimens.

**Fig. 3:**
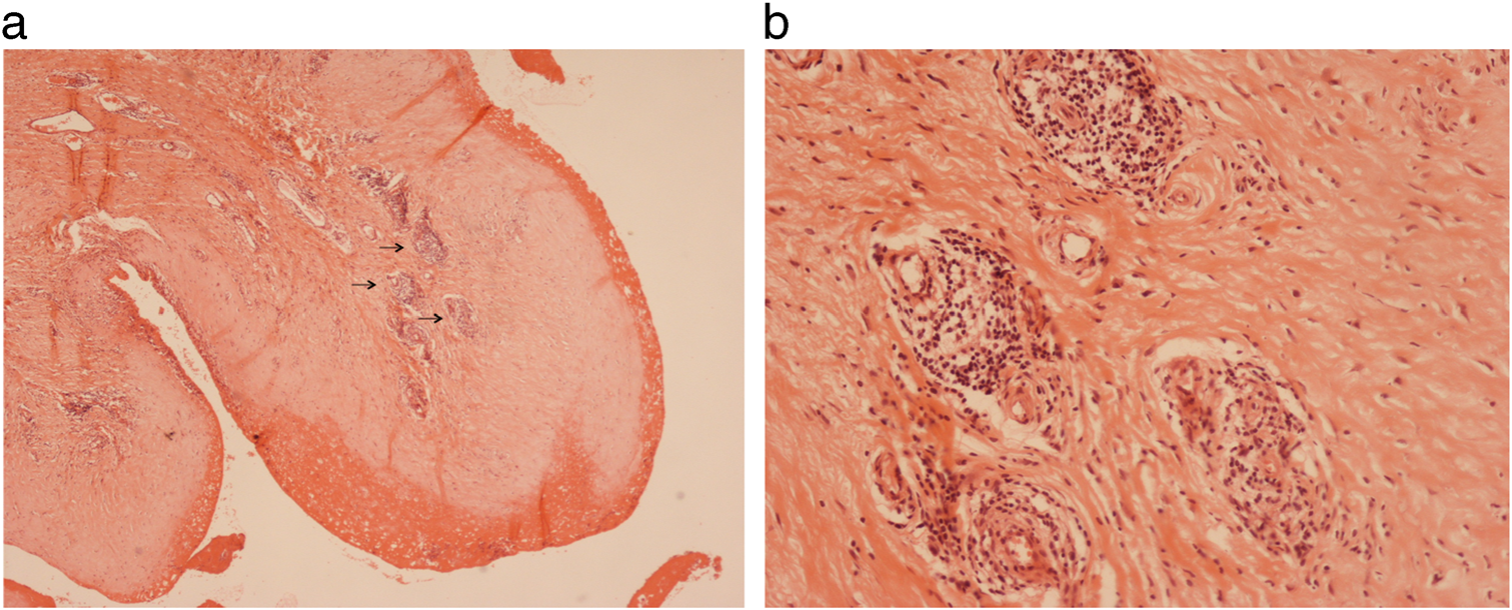
Histology of periprosthetic tissue membrane from a failed MF implant showing: **a** a zone of surface necrosis beneath which there are several perivascular lymphoid aggregates (some arrowed). **b** High power view of lymphoid aggregates in 3a

In all but two of the cases, the sampled periprosthetic tissues contained a scattered, focally heavy lymphocyte infiltrate (Table 1); plasma cells were seen in three cases. Eight cases (some of which were associated with relatively short implant duration) had a moderate or heavy lymphocyte infiltrate with Grade 2/3 Oxford ALVAL scores (Fig. 3). In nine cases the Oxford ALVAL score was Grade 1. There were three cases in which The Oxford ALVAL score was 0; in these cases, specimens of the capsule were composed entirely of necrotic tissue with only a few scattered macrophages and no obvious lymphocyte infiltrate.

**Fig. 4:**
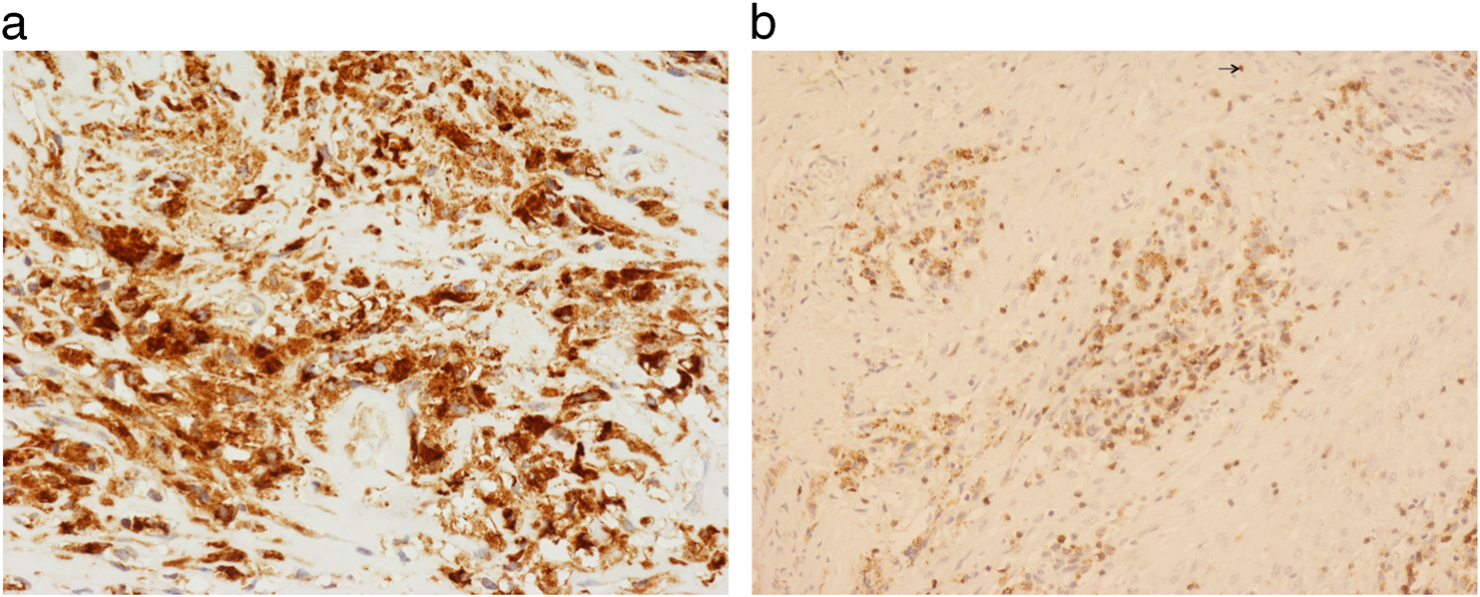
Immunohistochemistry of periprosthetic tissue membrane from a failed MF implant showing **a** staining of foreign body macrophages for CD68 (pan-macrophage marker) and **b** staining of perivascular lymphocytes for CD3 (T cell marker)

Immunohistochemistry showed that the mononuclear phagocytic cells containing metal wear particles strongly expressed the monocyte/macrophage markers CD14, CD68 (Fig. 4a) and CD163; these cells also strongly expressed HLA-DR. Lymphocytes in the perivascular lymphoid aggregates expressed the T-cell marker CD3 (Fig. 4b). A few cells also expressed the B-cell marker CD20. Most CD3+ T cells also expressed CD4, a marker of T-helper cells, with a minority of cells weakly expressing CD8, which is characteristic of T-cytotoxic/suppressor cells. Weak expression of the apoptosis marker bcl-2 was noted in macrophages and some lymphoid cells.

## Discussion

Although the MoM MF total hip prosthesis was characterised by a higher rate of early- and intermediate-term aseptic loosening than the MoP Charnley implant, a few studies reported similar rates of long-term implant loosening. Jacobsson et al. [9] reported a 20 year implant survival of 77% in 107 consecutive MF arthroplasties; this compared with 73% for a similar population with the Charnley prosthesis. Brown et al. [29] reported a 28 year MF implant survivorship of 74% and noted that the reported survival of MoM total hip arthroplasties was 53–89% at 10 to 15 years while that of the Charnley arthroplasty was 75–84% over 20 to 30 years. A review of short-term outcomes of the first 100 cases that received a MF hip implant in Oxford reported good or excellent results in 90% of cases [30]. A subsequent review comparing results of the MF and the Charnley total hip prosthesis in over 200 cases reported superior clinical outcomes and fewer complications with the Charnley implant [31]. Amstutz and Grigoris reviewed the clinical results of MF and other first- generation MoM hip implants and noted, as in the present study, some impressive outcomes [1].

The main cause of early MF hip implant failure was aseptic loosening. This complication was ascribed mainly to the MF implant being biomechanically at a disadvantage compared with the Charnley implant in design terms [1, 2, 5, 6]. A number of reports, however, noted pathological changes in periprosthetic tissues that were proposed as contributory factors to the development of implant loosening. Evans et al. [24] reported necrosis of bone and capsular tissue around MoM hip implants and noted a correlation with the severity and extent of obliterative changes in vessels; it was suggested that these changes occurred as a result of a sensitivity reaction to Co-Cr ions derived from metal wear particles. Winter [27] described necrosis in tissues around retrieved MoM hip replacements with particles deposited in acellular collagen and in phagocytic cells Jones et al. [26] also reported necrosis of bone and capsular tissue from retrieved first-generation MoM prostheses and attributed this to a toxic and hypersensitivity reaction to cobalt which he suggested caused an avascular phenomenon. The study of Brown et al. [22] noted significant tissue necrosis and a chronic inflammatory cell infiltrate in soft tissue and bone; metallic debris within cells and a prominent lymphocytic infiltrate with occasional plasma cells were also identified. Doorn et al. [23] and Howie et al. [25] also noted tissue necrosis and the presence of lymphocytes in retrieved periprosthetic tissues around MF and other first-generation MoM hip implants. Willert et al. [32], investigating (non-MF) failed first-generation MoM total hip arthroplasties, also noted a lymphocyte response in peri-implant tissues. Other studies allude to the presence of macrophages and giant cells, but do not mention tissue necrosis or a lymphoid infiltrate [7, 33–35].

The MF hip implant was composed of a similar Co–Cr alloy to that used in second- and third-generation MoM implants. In this study we show that periprosthetic tissues around MF implants exhibit similar pathological changes to those reported around modern MoM implants. Cell and tissue necrosis, a heavy infiltrate of macrophages in response to Co–Cr wear particles and ALVAL-like lymphoid aggregates, commonly seen in failed modern MoM hip implants [15–20, 36, 37], were also noted in failed MF hip implants with approximately 40% of cases exhibiting a Grade 2/3 ALVAL response.

The MoM articulation tends to produce less volumetric wear than other implant combinations such as MoP; however, the number of wear particles generated is an order of magnitude higher, the particles being much smaller and therefore much more numerous per unit volume [38, 39]. The nanometre-size Co–Cr particles induce a profound innate and adaptive immune response. Macrophage phagocytosis of metal wear particles is probably the major factor in causing the extensive necrosis in MoM periprosthetic tissues. Co–Cr particles are subject to corrosion and produce high levels of metal ions which are cytotoxic, causing apoptosis and necrosis of macrophages that release lysosomal enzymes into the tissues. Metal ions can also act as haptens, combining with cell and tissue proteins to induce a specific cell-mediated delayed hypersensitivity immune response. This is evidenced histologically in MoM implants by the presence of a predominantly T lymphocyte infiltrate which is often perivascular in nature [17, 36, 37]. T cells predominated in the lymphoid aggregates seen in failed MF cases, suggesting that the adaptive immune response to metal wear is similar.

The presence of profound necrotic and inflammatory changes in the MF periprosthetic tissues would indicate that Co–Cr wear particle-induced hypersensitivity and cytotoxicity are likely to have contributed, at least in part, to implant failure. A major limitation of our study is that, due to the many years that have elapsed, we do not have full clinical and radiological information on the cases we have analysed. Pseudotumours have been strongly associated with failure of modern MoM hip implants [15, 16, 19, 20] and, though not seen in our cases, there are two reports of probable pseudotumour-like lesions associated with MF implant failure [23, 40].

## References

[1] Amstutz HC, Grigoris P (1996). Metal on metal bearings in hip arthroplasty. Clin Orthop Relat Res.

[2] Cuckler JM (2005). The rationale for metal-on-metal total hip arthroplasty. Clin Orthop Relat Res.

[3] Knight SR, Aujla R, Biswas SP (2011). Total hip arthroplasty - over 100 years of operative history. Orthop Rev (Pavia).

[4] McKee GK, Watson-Farrar J (1966). Replacement of arthritic hips by the McKee-Farrar prosthesis. J Bone Joint Surg Br.

[5] Schmalzried TP, Szuszczewicz ES, Akizuki KH, Petersen TD, Amstutz HC (1996). Factors correlating with long term survival of McKee-Farrar total hip prostheses. Clin Orthop Relat Res.

[6] Walker PS, Salvati E, Hotzler RK (1974). The wear on removed McKee-Farrar total hip prostheses. J Bone Joint Surg Am.

[7] Clarke MT, Darrah C, Stewart T, Ingham E, Fisher J, Nolan JF (2005). Long-term clinical, radiological and histopathological follow-up of a well-fixed Mckee-Farrar metal-on-metal total hip arthroplasty. J Arthroplasty.

[8] Jacobsson SA, Djerf K, Wahlstrom O (1990). A comparative study between McKee-Farrar and Charnley arthroplasty with long-term follow-up periods. J Arthroplasty.

[9] Jacobsson SA, Djerf K, Wahlstrom O (1996). Twenty-year results of McKee-Farrar versus Charnley prosthesis. Clin Orthop Relat Res.

[10] Jantsch S, Schwagerl W, Zenz P, Semlitsch M, Fertschak W (1991). Long-term results after implantation of McKee-Farrar total hip prostheses. Arch Orthop Trauma Surg.

[11] Amstutz HC, Campbell P, McKellop H, Schmalzreid TP, Gillespie WJ, Howie D, Jacobs J, Medley J, Merritt K (1996). Metal on metal total hip replacement workshop consensus document. Clin Orthop Relat Res.

[12] de Steiger RN, Hang JR, Miller LN, Graves SE, Davidson DC (2011). Five-year results of the ASR XL acetabular system and the ASR hip resurfacing system: an analysis from the Australian orthopaedic association national joint replacement registry. J Bone Joint Surg Am.

[13] Langton DJ, Jameson SS, Joyce TJ, Gandhi JN, Sidaginamale R, Mereddy P, Lord J, Nargol AV (2011). Accelerating failure rate of the ASR total hip replacement. J Bone Joint Surg Br.

[14] Langton DJ, Jameson SS, Joyce TJ, Hallab NJ, Natu S, Nargol AV (2010). Early failure of metal-on-metal bearings in hip resurfacing and large-diameter total hip replacement: a consequence of excess wear. J Bone Joint Surg Br.

[15] Bauer T. What does the histology tell us? In: Jones LC, Greenwold AS, Haggard WO, editors. Metal-on-metal bearing. A clinical practicum. New York, Springer; 2013. P. 153–62.

[16] Grammatopoulos G, Pandit H, Kamali A, Maggiani F, Glyn-Jones S, Gill HS, Murray DW, Athanasou N (2013). The correlation of wear with histological features after failed hip resurfacing arthroplasty. J Bone Joint Surg Am.

[17] Mahendra G, Pandit H, Kliskey K, Murray D, Gill HS, Athanasou N (2009). Necrotic and inflammatory changes in metal-on-metal resurfacing hip arthroplasties. Acta Orthop.

[18] Natu S, Sidaginamale RP, Gandhi J, Langton DJ, Nargol AV (2012). Adverse reactions to metal debris: histopathological features of periprosthetic soft tissue reactions seen in association with failed metal on metal hip arthroplasties. J Clin Pathol.

[19] Pandit H, Glyn-Jones S, McLardy-Smith P, Gundle R, Whitwell D, Gibbons CL, Ostlere S, Athanasou N, Gill HS, Murray DW (2008). Pseudotumours associated with metal-on-metal hip resurfacings. J Bone Joint Surg Br.

[20] Campbell P, Ebramzadeh E, Nelson S, Takamura K, De Smet K, Amstutz HC (2010). Histological features of pseudotumour-like tissues from metal-on-metal hips. Clin Orthop Relat Res.

[21] Watters TS, Cardona DM, Menon KS, Vinson EN, Bolognesi MP, Dodd LG (2010). Aseptic lymphocyte-dominated vasculitis-associated lesion: a clinicopathologic review of an underrecognized cause of prosthetic failure. Am J Clin Pathol.

[22] Brown GC, Lockshin MD, Salvati EA, Bullough PG (1977). Sensitivity to metal as a possible cause of sterile loosening after cobalt-chromium total hip-replacement arthroplasty. J Bone Joint Surg Am.

[23] Doorn PF, Mirra JM, Campbell PA, Amstutz HC (1996). Tissue reaction to metal on metal total hip prostheses. Clin Orthop Relat Res.

[24] Evans EM, Freeman MA, Miller AJ, Vernon-Roberts B (1974). Metal sensitivity as a cause of bone necrosis and loosening of the prosthesis in total joint replacement. J Bone Joint Surg Br.

[25] Howie DW (1990). Tissue response in relation to type of wear particles around failed hip arthroplasties. J Arthroplasty.

[26] Jones DA, Lucas HK, O’Driscoll M, Price CH, Wibberley B (1975). Cobalt toxicity after McKee hip arthroplasty. J Bone Joint Surg Br.

[27] Winter G (1974). Tissue recation to metallic wear and corrosion products in human patients. J Biomed Materials Res.

[28] Phillips EA, Klein GR, Cates HE, Kurtz SM, Steinbeck M (2014). Histological characterization of periprosthetic tissue responses for metal-on-metal hip replacement. J Long Term Eff Med Implants.

[29] Brown SR, Davies WA, DeHeer DH, Swanson AB (2002). Long-term survival of McKee-Farrar total hip prostheses. Clin Orthop Relat Res.

[30] Somerville EW, Bentley GJ. McKee-Farrar total hip replacement arthroplasty. a review of the first 100 cases at the nuffield orthopaedic centre. Orthopaedics Oxford 1970;3:73–7.

[31] Bentley G, Duthie RB (1973). A comparative review of the McKee-Farrar and Charnley total hip prostheses. Clin Orthop Relat Res.

[32] Willert HG, Buchhorn GH, Gobel D, Koster G, Schaffner S, Schenk R, Semlitsch M (1996). Wear behavior and histopathology of classic cemented metal on metal hip endoprostheses. Clin Orthop Relat Res.

[33] Higuchi F, Inoue A, Semlitsch M (1997). Metal-on-metal CoCrMo McKee-Farrar total hip arthroplasty: characteristics from a long-term follow-up study. Arch Orthop Trauma Surg.

[34] Schmalzried TP, Peters PC, Maurer BT, Bragdon CR, Harris WH (1996). Long-duration metal-on-metal total hip arthroplasties with low wear of the articulating surfaces. J Arthroplasty.

[35] Zahiri CA, Schmalzried TP, Ebramzadeh E, Szuszczewicz ES, Salib D, Kim C, Amstutz HC (1999). Lessons learned from loosening of the McKee-Farrar metal-on-metal total hip replacement. J Arthroplasty.

[36] Davies AP, Willert HG, Campbell PA, Learmonth ID, Case CP (2005). An unusual lymphocytic perivascular infiltration in tissues around contemporary metal-on-metal joint replacements. J Bone Joint Surg Am.

[37] Willert HG, Buchhorn GH, Fayyazi A, Flury R, Windler M, Koster G, Lohmann CH (2005). Metal-on-metal bearings and hypersensitivity in patients with artificial hip joints. A clinical and histomorphological study. J Bone Joint Surg Am.

[38] Hallab NJ, Jacobs JJ (2009). Biological effects of implant debris. Bull NYU Hospit Jt Dis.

[39] Athanasou NA (2016). The pathobiology and pathology of aseptic implant failure. Bone Joint Res.

[40] Madan S, Jowett RL, Goodwin MI (2000). Recurrent intrapelvic cyst complicating metal-on-metal cemented total hip arthroplasty. Arch Orthop Trauma Surg.

